# Standing cough test stratification of moderate male stress urinary incontinence

**DOI:** 10.1590/S1677-5538.IBJU.2020.0551

**Published:** 2021-02-03

**Authors:** Roger K. Khouri, Yooni A. Yi, Nicolas M. Ortiz, Adam S. Baumgarten, Ellen E. Ward, Maia E. VanDyke, Steven J. Hudak, Allen F. Morey

**Affiliations:** 1 University of Texas Southwestern Medical Center Department of Urology Dallas Texas USA Department of Urology, University of Texas Southwestern Medical Center, Dallas, Texas, USA.

**Keywords:** Urinary Sphincter, Artificial, Suburethral Slings, Suburethral Slings

## Abstract

**Purpose:**

Patient-reported history of pads per day (PPD) is widely recognized as a fundamental element of decision-making for anti-incontinence procedures. We hypothesize that SUI severity is often underestimated among men with moderate SUI. We sought to compare patient history of incontinence severity versus objective in-office physical examination findings.

**Materials and Methods:**

We retrospectively reviewed our single-surgeon male SUI surgical database from 2007-2019. We excluded patients with incomplete preoperative or postoperative data and those who reported either mild or severe SUI, thus having more straightforward surgical counseling. For men reported to have moderate SUI, we determined the frequency of upgrading SUI severity by recording the results of an in-office standing cough test (SCT) using the Male Stress Incontinence Grading Scale (MSIGS). The correlation of MSIGS with sling success rate was calculated. Failure was defined as >1 PPD usage or need for additional incontinence procedure.

**Results:**

Among 233 patients with reported moderate SUI (2-3 PPD), 89 (38%) had MSIGS 3-4 on SCT, indicating severe SUI. Among patients with 2-3 PPD preoperatively, sling success rates were significantly higher for patients with MSIGS 0-2 (76/116, 64%) compared to MSIGS 3-4 (6/18, 33%) (p <0.01).

**Conclusions:**

Many men with self-reported history of moderate SUI actually present severe SUI observed on SCT. The SCT is a useful tool to stratify moderate SUI patients to more accurately predict sling success.

## INTRODUCTION

Stress urinary incontinence (SUI) persists long-term in approximately 20% of patients after radical prostatectomy and 10% of patients after prostate radiation (1–4). Men with mild SUI are ideal candidates for transobturator slings (5–8), while men with severe SUI are better suited for artificial urinary sphincters (AUS) (9–11). Men with moderate SUI, however, are often considered to be candidates for either sling or AUS (6, 7, 12, 13). Counseling patients with moderate SUI poses a challenge because of the lack of a validated method of prognostication (12, 14).

Although pad per day (PPD) measurements allow for an estimation of incontinence severity, variation in activity, type of pad used, and degree of soiling before switching pads create uncertainty. Given the emphasis on the physical exam in the 2019 AUA guidelines on surgical management of both male and female SUI (6, 15), we incorporated the standing cough test (SCT) into our standard evaluation of male SUI. The SCT has been validated as a reproducible and reliable test for grading male SUI (16), and it has been shown to correlate strongly with 24-hour pad-weight (17).

Many patients reported as having moderate SUI are referred to our practice specifically for sling placement due to its perceived simplicity versus AUS. We hypothesize that a substantial proportion of men reported to have moderate SUI will have severe leakage on SCT and thus would be better served with AUS. We sought to determine the percentage of men with a history of moderate SUI who were actually found to have severe leakage on SCT, and we analyzed the utility of SCT in stratifying these men in terms of success after sling surgery. To our knowledge, this is the first study to compare patient reported SUI severity to observed SUI severity on SCT.

## MATERIALS AND METHODS

We retrospectively reviewed our single-surgeon male SUI surgical database from 2007-2019 and identified patients who underwent AUS or AdVance sling placement (IRB: STU-102012019). Early in our experience, we did not consistently obtain preoperative Male Stress Incontinence Grading System (MSIGS) data according to our current protocol. We excluded patients with incomplete preoperative MSIGS or post-operative follow-up data. SUI severity was defined by the preoperative pads per day (PPD) usage: 0-1 PPD=mild; 2-3 PPD=moderate; ≥4 PPD=severe; these definitions are based on our prior study on risk factors for sling failure (18). For this sub-analysis, we excluded patients with mild and severe SUI and focused solely on the patients with moderate SUI. There were no other exclusion criteria. All other patients in our database of adult men undergoing AUS or sling for SUI were included in the analysis.

All patients underwent SCT to physically demonstrate the degree of SUI during clinic evaluation. Patients verbally confirmed that they had not voided for at least one hour prior to the SCT to ensure presence of urine in the bladder. The examiner evaluated the urethral meatus while the patient performed a series of four forceful coughs. The degree of leakage was scored using the Male Stress Incontinence Grading Scale (MSIGS, Appendix-1).

Among the patients with reported moderate SUI, we used their preoperative MSIGS to stratify them into favorable (MSIGS 0-2) and unfavorable (MSIGS 3-4). MSIGS scores of 3-4 were considered unfavorable, as it suggests a high degree of sphincteric incompetence. The patients with favorable moderate SUI who underwent sling placement (Group-A) were compared to the patients with unfavorable moderate SUI who underwent sling placement (Group-B). Sling failure was defined as >1 PPD usage or need for additional incontinence procedure. Preoperative and postoperative data were compared between Groups A and B with two-tailed, unpaired t-tests for continuous variables and chi-square tests for categorical variables. Statistical analyses were performed using Python 3.0 with p <0.05 considered statistically significant.

## RESULTS

**Figure 1:**
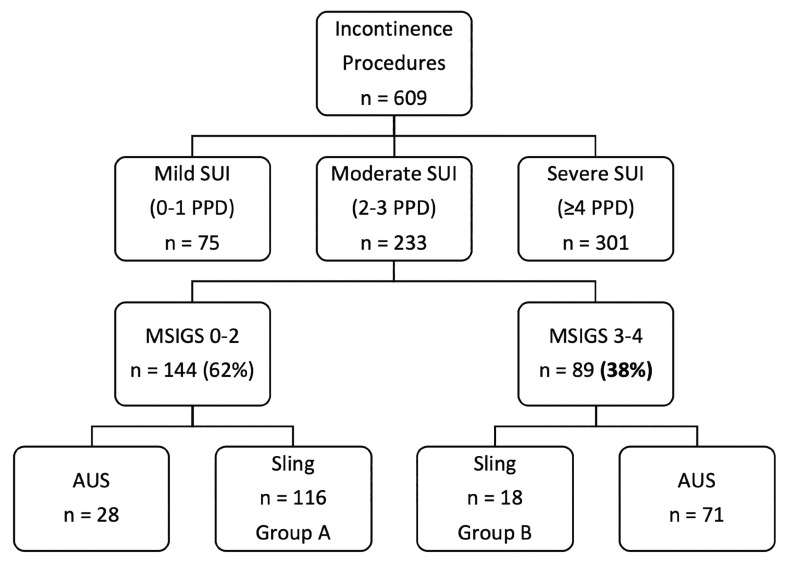
Flow chart. Patients with moderate SUI were stratified into favorable and unfavorable, depending on Male Stress Incontinence Grading System (MSIGS) score.

Among 978 SUI cases, we excluded 369 who had incomplete preoperative or postoperative data. Another 376 men were excluded because they reported either mild or severe SUI, thus having a more straightforward surgical counseling (towards sling or AUS, respectively). Among the 233 selected remaining patients presenting with moderate SUI who comprised the study cohort, 144 (62%) were deemed favorable by SCT (MSIGS 0-2, Figure-1), while more than one-third (89/233, 38%) were observed to have an unfavorable, severe degree of stress-induced urinary leakage during in-office examination (MSIGS 3-4).

**Figure 2:**
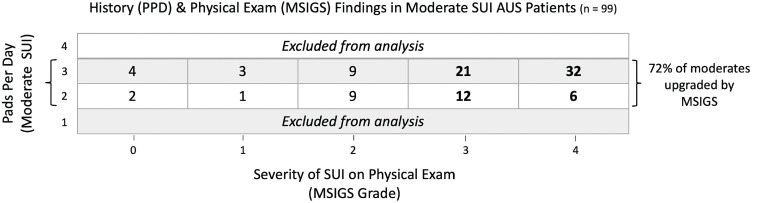
Relationship between preoperative PPD usage and Male Stress Incontinence Grading System (MSIGS) grade among AUS patients with reported moderate SUI.

Group-A was comprised of the 116 men with favorable moderate SUI who underwent sling placement. Of the 89 men (38%) with unfavorable moderate SUI, 18 underwent sling placement (Group-B). At a mean follow-up of 31.3 months, Group-A had a significantly higher success rate (76/116, 64%) than Group-B (6/18, 33%) (p <0.01) (Table-1). There was no difference in the complication rate between Group-A (20/116, 17%) and Group-B (4/18, 22%) (p=0.61). Of the 99 AUS patients with moderate SUI, over 70% were upgraded based on demonstrated severe levels of stress-induced leakage and unfavorable MSIGS grade (Figure-2).

**Table 1:** Preoperative demographics and clinical history and postoperative outcomes among men with reported moderate SUI (2-3 PPD), stratified by favorable (MSIGS 0-2) and unfavorable (MSIGS 3-4).

		MSIGS 0-2 (Group A)	MSIGS 3-4 (Group B)	p Value
No. total patients		116	18	
Mean Baseline PPD (SD)		2.23 (0.65)	2.53 (0.81)	0.15
Mean MSIGS (SD)		1.34 (0.79)	3.29 (0.57)	**<0.01**
Mean age at sling (SD)		67.6 (7.9)	64.3 (8.4)	0.07
Mean BMI (SD)		27.7 (3.9)	26.6 (4.0)	0.53
**No. comorbidities (%)**				
	Hypertension	63 (54.3%)	10 (55.6%)	0.92
	Diabetes mellitus	15 (12.9%)	5 (27.8%)	0.10
	Tobacco history	62 (53.4%)	8 (44.4%)	0.48
**No. urological history (%)**				
	Prostate surgery	113 (97.4%)	16 (88.9%)	0.08
	Prostate radiation	15 (12.9%)	2 (11.1%)	0.83
	Androgen deprivation	3 (2.6%)	1 (5.6%)	0.49
	Neurogenic bladder	0	0	1.00
	Prior sling	3 (2.6%)	1 (5.6%)	0.49
	ED	78 (67.2%)	11 (61.1%)	0.61
	Prior IPP	6 (5.2%)	1 (5.6%)	0.95
	Concurrent IPP placement	28 (24.1%)	2 (11.1%)	0.22
	No. complications (%)	20 (17.2%)	4 (22.2%)	0.61
	No. sling success (%)	76 (63.9%)	6 (33.3%)	**<0.01**

**SUI** = stress urinary incontinence; **MSIGS** = male stress incontinence grading scale; **PPD** = pads per day; **SD** = standard deviation; **BMI** = body mass index; **ED** = erectile dysfunction; **IPP** = inflatable penile prosthesis

## DISCUSSION

Current guidelines state that men with moderate SUI are candidates for either an AUS or a transobturator sling, but there are no established protocols for further stratifying these patients (6, 12, 13, 19). In this study, we found that almost 40% of men who reported moderate SUI demonstrated severe leakage on SCT, suggesting that subjective assessment of SUI is inadequate to fully characterize SUI severity. Furthermore, stratifying men who reported moderate SUI into favorable or unfavorable categories based on MSIGS values allowed for accurate prediction of sling success.

It is important to note that many of the men in this study were referred to our practice specifically for urethral sling placement for moderate SUI-in this cohort, a clear discordance was often noted between reported history and physical exam findings. MSIGS allowed us to easily and more precisely assess SUI severity and counsel patients on surgical options accordingly.

### Male SUI Assessment

Prior studies have demonstrated that pre-operative 24-hour pad weight, PPD usage, and MSIGS scores are all predictors of sling success (5, 16–21). PPD usage is a simple way to estimate SUI severity. However, PPD usage is difficult to accurately quantify because of variations in the type of pad used, patient activity level, and the degree of wetting before switching pads. The 24-hour pad weight is a reproducible method to quantify SUI and correlates well with surgical outcomes (12, 22). However, many patients find the 24-hour pad weight tedious and cumbersome, and many forget to collect all of their pads for a full 24-hour period or to bring the pads in for their clinic visits. These challenges have limited the widespread adoption of 24-hour pad weights (23). While more invasive tests, such as cystoscopy and urodynamics, may provide more detailed information on the anatomy and function of the lower urinary tract, they have much higher cost and time requirements than SCT. Moreover, their utility in predicting sling success has not been established (2, 20, 24, 25).

In 1996, Kowalcyzk and colleagues first described the SCT as a method to stratify male SUI severity to determine if one or two AUS cuffs should be used (26). We no longer perform tandem cuff AUS procedures, but we have incorporated the SCT as a method to stratify SUI severity for many years (16, 17). The SCT is an easy and reproducible in-office assessment of SUI severity that provides prognostic value in the evaluation of men with moderate SUI who are considering incontinence surgery. It also correlates strongly with 24-hour pad weights (17). Patients found to have unfavorable moderate SUI could then be informed of the low success rate of sling and encouraged to consider an AUS. We have previously demonstrated that the SCT adds predictive value for sling success for patients who already have PPD and 24-hour pad weight measurements (18).

Also, we have previously demonstrated that each point increase in MSIGS from 0-4 has an approximately equal effect on the probability of sling success (18). In practice, authors should not overemphasize the difference between MSIGS2 and MSIGS3, but rather, consider MSIGS as a continuous variable in the context of all the other available data when discussing management options with patients. The distinction between MSIGS2 and MSIGS3 is the most clinically relevant, however, because MSIGS1 and MSIGS4 are more straightforward to manage.

The value of MSIGS lies in its practicality; it is a non-invasive test that incurs no additional costs. Other variables with no invasiveness or cost, such as demographics and general clinical data have not been shown to predict SUI severity or sling success. More invasive and expensive tests, such as sphincter pressure under contraction (SPUC) have demonstrated clinical utility (28); however, more studies on SPUC are needed to show that its benefits outweigh the costs and invasiveness. This study supports our recent findings that MSIGS is a highly practical and important variable when predicting surgical outcomes of an AUS or sling (27).

### Limitations

This study has several limitations. The single-surgeon design might limit generalizability. However, the surgical techniques and clinical strategies used in this study closely follow standard practice guidelines and are likely similar to those at other institutions. The retrospective study design could allow for confounding; however, there were no significant differences in preoperative variables between groups A and B. Also, while there is no established definition of moderate SUI in terms of PPD, we used a conservative definition of 2-3 PPD. If we had defined moderate SUI as 3-5 PPD, we would have found a much higher rate of upstaging with MSIGS. This highlights the need to objectively grade SUI severity with MSIGS.

Our study is also limited by the absence of adjustable devices for mild to moderate SUI. The adjustable transobturator male sling (ATOMS), Remeex, ProACT, and ARGUS slings have all demonstrated safety and efficacy (29, 30). These adjustable slings may prove to have a greater role in the moderate SUI population; however, additional studies will be needed comparing these devices to AUS and retropubic slings for men with varying degrees of SUI.

Pre-operative SUI severity and post-operative treatment success could have been more comprehensively assessed with 24-hour pad weight data. However, our patients find the 24-hour urine collection tedious and cumbersome. Often times, many forget to collect pads for a full 24-hour period, or they forget to bring the pads in for their clinic visits. Since the SCT correlates strongly with 24-hour pad weights (17) and has significant workflow limitations, we do not typically include 24-hour pad weights in our assessment. Finally, while formal urodynamic studies would add additional objective assessment of preoperative SUI severity and treatment outcomes, this study aimed to assess the utility of the more practical SCT to facilitate risk stratification and surgical decision making for moderate male SUI.

## CONCLUSIONS

Many men who report moderate SUI are found to actually have severe SUI observed during in-office physical examination. Our findings suggest that sling success can be predicted by stratifying men into favorable or unfavorable MSIGS groups using the Standing Cough Test.

## ABBREVIATIONS

SUI: stress urinary incontinence
AUS: artificial urinary sphincter
PPD: Pads per day
MSIGS: Male Stress Incontinence Grading Scale
SCT: Standing cough test

## APPENDIX

**Appendix 1:** Male Stress Incontinence Gradina Scale (MSIGS).

Grade	Definition
0	No leakage
1	Delayed drops only
2	Early drops, no stream
3	Early drops, delayed stream
4	Early and persistent stream
